# Network Conduciveness with Application to the Graph-Coloring and Independent-Set Optimization Transitions

**DOI:** 10.1371/journal.pone.0011232

**Published:** 2010-07-08

**Authors:** Valmir C. Barbosa

**Affiliations:** Programa de Engenharia de Sistemas e Computação, Instituto Alberto Luiz Coimbra de Pós-Graduação e Pesquisa de Engenharia (COPPE), Universidade Federal do Rio de Janeiro, Rio de Janeiro, Brazil; University of East Piedmont, Italy

## Abstract

**Background:**

Given an undirected graph, we consider the two problems of combinatorial optimization, which ask that its chromatic and independence numbers be found. Although both problems are NP-hard, when either one is solved on the incrementally denser graphs of a random sequence, at certain critical values of the number of edges, it happens that the transition to the next value causes optimal solutions to be obtainable substantially more easily than right before it.

**Methodology/Principal Findings:**

We introduce the notion of a network's conduciveness, a probabilistically interpretable measure of how the network's structure allows it to be conducive to roaming agents, in certain conditions, from one portion of the network to another. We demonstrate that the performance jumps of graph coloring and independent sets at the critical-value transitions in the number of edges can be understood by resorting to the network that represents the solution space of the problems for each graph and examining its conduciveness between the non-optimal solutions and the optimal ones. Right past each transition, this network becomes strikingly more conducive in the direction of the optimal solutions than it was just before it, while at the same time becoming less conducive in the opposite direction.

**Conclusions/Significance:**

Network conduciveness provides a useful conceptual framework for explaining the performance jumps associated with graph coloring and independent sets. We believe it may also become instrumental in helping clarify further issues related to NP-hardness that remain poorly understood. Additionally, it may become useful also in other areas in which network theory has a role to play.

## Introduction

The past decade has seen an impressive growth in the science of complex networks, understood as the branch of scientific inquiry which, by merging well established notions and techniques from the theory of graphs and from statistical physics, addresses the interplay of structure and function in the large, essentially unstructured networks that occur in a wide variety of domains. The latter have encompassed several instances in many biological, social, and technological fields, and have yielded an equally variegated array of results that the reader can now refer to in books and paper collections such as [1]–[3].

One common methodological thread in all these studies has been the definition of a graph to represent the interactions among certain entities in the domain of interest, followed by the analysis of mathematical descriptors of some of the graph's properties as averages over a number of graphs generated according to some random-graph model thought to represent the phenomenon under consideration. Thus have emerged important finds regarding the characterization of some networks as small-world structures, or as scale-free structures, as well as powerful structural indicators of a graph's nature, such as its clustering coefficient and various centrality-related quantities.

Here we introduce another indicator of a graph's properties, called its conduciveness. Given a directed graph 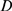 of node set 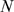, the conduciveness of 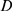 is defined with respect to two subsets 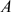 and 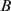 of 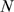. Let 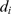 denote the out-degree of node 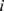 in 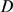 (the node's number of outgoing edges) and define 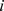
's 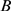-bound out-degree, denoted by 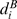, to be its number of outgoing edges whose heads are members of 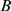 (i.e., edges that lead from 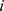 to some member of 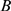). The conduciveness of 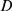 from 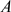 to 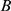 is denoted by 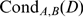 and given by
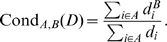
(1)Clearly, 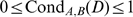.

This definition of a directed graph's conduciveness can be easily interpreted in the context of hypothetical agents inhabiting the graph at its nodes but free to roam to other nodes by taking steps that follow edges along their directions. Specifically, 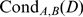 is the probability that, conditioned on there being one agent at each and every node in 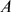, one further random step out of all those that the agents can take leads to a node in 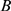. A graph for which this probability is higher than for another is regarded as more conducive to set 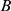 from set 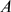 than that other graph.

Our initial motivation for the introduction of this definition has been its potential application to explain some phenomena related to the complexity of solving certain problems of combinatorial optimization. Normally such a problem is defined on the set 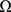 of the feasible solutions to the problem, using a real function 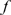 defined over 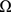 according to which an optimal member of 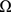 is to be found (one for which 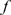 is minimum or maximum over all of 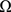, depending on the problem). Many such problems are NP-hard, meaning that finding optimal solutions to them is at least as hard as solving any of the decision problems that constitute the class NP (those whose solutions, should they somehow be provided at no cost and turn out to be affirmative, could be checked to be correct in polynomial time [4]). That no polynomial-time deterministic algorithm has ever been found to solve an NP-hard problem is normally taken as a sign of computational intractability as problem instances grow large.

This, however, is to be taken with caution. The class NP can be viewed as a complex hierarchy of subclasses [5], which may ultimately help account for what is observed in practice: some NP-hard problems are solvable much more efficiently than others; more strikingly, two similarly sized instances of the same NP-hard problem may require considerably different amounts of computational effort to be solved. A way to illustrate this that is useful in the context of this paper is based on the following. Let there be 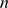 nodes and, for 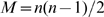, consider a sequence 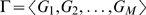 of undirected graphs on these nodes. For 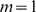, 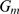 has one single edge joining two randomly chosen nodes and 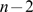 isolated nodes. For 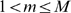, 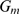 is obtained from 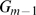 by placing a further edge between two randomly chosen nodes that are not already joined by an edge; so 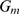 has 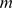 edges and, for relatively small 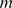, may also have isolated nodes.

The crucial observation is that, as first documented in [6], [7] in the wake of what was done earlier for some NP-hard decision problems [8]–[12], there exist NP-hard optimization problems for which practically every attempted algorithm, deterministic or otherwise, undergoes sharp performance variations when applied to the graphs in 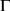 for increasing values of 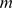. These variations refer to jumps in how long it takes the algorithm to reach an optimal solution and happen at well-defined critical values of 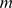 given 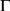. The same initial reporters of these phenomena also offered tentative explanations related to the nature and structure of the corresponding 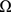 sets (one for each of the 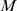 graphs in 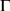), but those have lacked full consistency owing to normalization difficulties as the sizes of those sets grow along with 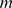 [7]. It has also been a difficulty that the values of 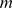 at which the jumps occur tend to be different if 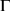 is changed, so the aforementioned analyses have only addressed single graph sequences and therefore lack statistical significance as well.

We have found that the notion of a directed graph's conduciveness, as introduced above, has an important role to play in elucidating the nature of these performance jumps. The fundamental idea is, for each 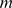, first to identify an appropriate descriptor of the feasible solutions to the problem that is being posed on 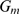. This will give us the 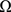 set for that particular 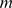, henceforth denoted by 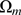. Then we identify some primitive operation on the members of 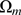 that may be used to transform one of them into another. Every two members of 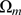 that are thus related constitute an ordered pair; collectively, all such pairs constitute the set that we denote by 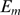. The directed graph whose conduciveness we study, denoted by 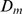, has node set 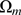 and edge set 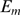. This graph embodies all primitive steps that an optimum-seeking algorithm may take to solve the problem on 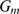. For 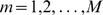, we study the conduciveness of 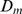 from the nodes in 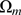 that do not represent optimal solutions to those that do.

By its very nature as a probability, this conduciveness of 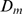 has none of the normalization problems alluded to above. As we will see, it also allows for some multiplicity of events to be investigated for statistical significance, though only to a limited extent. This is because in general the edges of 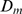 can only be found through the explicit enumeration and testing of several pairs of members of 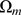, which in most cases is a very large set even for very small values of 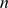. We then see that there exist severe time constraints on the generation of the 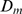 graphs for multiple instances of the sequence 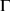, and consequently constraints on the largest value of 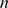 that can realistically be used. We note, moreover, that seldom can 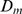 be fully stored, which limits the properties that can be analyzed.

We target the same two optimization problems as [7], namely the problem of coloring the nodes of an undirected graph optimally and that of finding one of its maximum independent sets. Aside from the fact that they are both paradigmatic NP-hard optimization problems, our choice of them has also been influenced by the remarkable fact that, for each value of 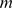, it is possible to define a single 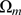 set for both problems, thus allowing the study of their performance jumps to be conducted in a peculiarly interrelated fashion.

## Methods

The chromatic number of an undirected graph 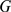 on 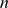 nodes, denoted by 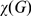, is an integer between 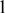 and 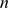 indicating the smallest number of distinct colors (labels) that can be used to tag the nodes of 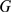 in such a way that every node gets exactly one color and no two nodes connected by an edge get the same color. The independence (or stability) number of 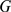, denoted by 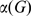, is likewise an integer between 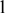 and 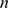 and indicates the size of a largest independent subset of 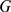
's node set, that is, a largest subset of nodes containing no two nodes connected by an edge [13]. Finding either number is an NP-hard problem [4].

The two problems can be reformulated in such a way that their sets 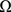 of feasible solutions are in fact the same set. To see this, first let an orientation of 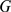 be an assignment of directions to 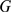
's edges, that is, one of the ways in which 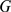 can be turned into a directed graph. An orientation is acyclic if it contains no directed cycles (i.e., it is never possible to return to a node after moving away from it along the directions of the edges). Every acyclic orientation of 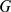 yields a number of colors to tag the nodes of 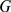 legitimately, and likewise an independent set of 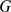. Conversely, every legitimate assignment of colors to the nodes of 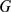 yields an acyclic orientation of 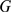, and so does every independent set of 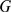. The proofs that back up these statements are not simple [14], but accepting them clearly implies that both finding 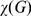 and finding 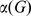 can be formulated based on sharing the 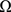 set defined as the set of all the acyclic orientations of 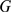.

The precise relationships implied by the proofs in [14] are the following. Let 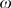 be an acyclic orientation of 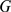. Let 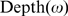 denote the number of nodes on a longest directed path in 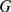 according to 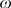, henceforth referred to simply as the depth of 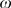. Then
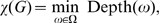
(2)that is, 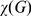 is the depth of the shallowest member of 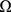. Now let 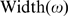 denote the least number of node-disjoint directed paths into which 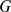 can be decomposed given 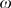, henceforth referred to simply as the width of 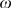. Then
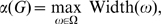
(3)meaning that 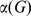 is the width of the widest member of 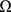.

It also emerges from those same proofs (but see [15], [16] for explicit accounts of the corresponding algorithms) that, given 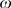, both 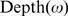 and 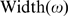 can be computed in polynomial time. So, by Equations (2) and (3), the NP-hardness of the two problems in question is to be attributed to the inherent difficulty of searching inside 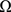 for an optimal 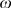 in each case. Following the general outline provided in the previous section, we continue by defining the directed graph 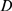 of node set 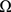 whose edge set, 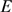, is to be set up to reflect some primitive relationship among the members of 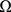 that can be used to transform each one into some other.

There are certainly several ways in which an acyclic orientation, say 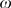, can be turned into another, say 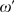. One possibility that has become popular in several task scheduling applications is to turn one or more of the sinks of 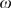 (nodes with no outgoing edges) into sources (nodes with no incoming edges) and then let the resulting orientation be 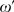 (clearly acyclic, given the acyclicity of 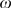). We eschew this choice for two reasons. The first one is that it entails several direction reversals for one single transformation, which then seems hard to qualify as primitive. The second reason is that, under such sink-to-source transformations, the resulting 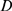 is almost always a fragmented graph (i.e., there exist pairs of nodes that are unreachable from each other even if edge directions are ignored) [17]. Since our interest is in the conduciveness of 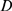 with respect to certain subsets of 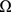, it seems that starting out with a fragmented 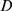 is bound to produce results somewhat devoid of meaning.

Our definition of the edge set 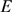 of 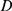 is then the following. Given 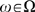, an edge exists directed from 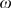 to some 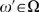 if and only if 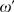 results from reversing the direction of exactly one of the edges of 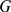 as oriented by 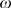. Of course, if 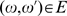 holds, then so does 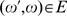. Moreover, it now holds that 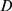 is strongly connected, that is, a directed path exists from any node to any other. (To see that the latter holds, consider that, if it does not, then there have to exist 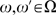 with the following property. If 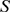 is the set of edges in 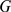 on whose directions 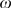 and 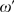 disagree, then the individual direction reversal of any edge 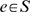 creates a directed cycle 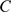 in the resulting orientation. But since 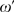 is acyclic, 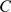 must also contain another of the edges of 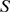, say 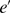, and this edge's direction must oppose that of 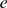 on 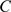. Notice, however, that 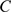 comprises at least three edges, so reversing the direction of 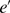 alone would create no directed cycle. This contradicts the existence of the 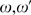 pair with the assumed property.)

Handling 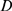 computationally, though, is a difficult matter owing to both its number of nodes and the explicit way in which its edges must be enumerated. The number of nodes, which is the number of distinct acyclic orientations of 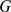, is given by a surprising application of the so-called chromatic polynomial of 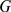 [18] and, for 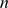 fixed, grows rapidly from the two orientations allowed by the case of one single edge to the 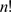 orientations that a graph with all possible 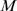 edges on 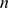 nodes admits. As for discovering the edges of 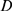 that outgo from a particular orientation 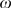, there is in general no alternative but to try and reverse the directions of all edges of 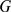, one by one with respect to what 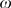 stipulates, checking for each one whether the resulting orientation is itself a member of 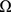.

Given 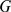, we enumerate the members of 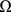 by the algorithm given in [19] but store each one only while recording some of its properties for later use. For each 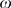 that is output by the algorithm, we calculate 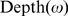, 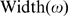, its out-degree 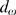 in 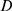, and its 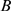-bound out-degree 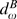. Here 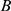 depends on which problem is being addressed. If it is the coloring problem, then 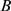 is the subset of 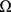 comprising orientations whose depths are all equal to 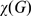. If it is the independent-set problem, then 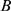 is the subset of 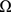 whose orientations all have width 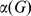. For simplicity, whenever the context allows we refer to 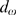 as a full degree and to 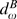 as an optimum-bound degree. Note that each full degree is an integer between 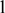 and the number of edges of 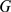. An optimum-bound degree, in turn, is an integer between 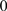 and again the number of edges of 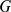.

Scarce though they may be, these recorded properties of 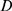 allow for some useful statistics to be computed, in addition to allowing for the direct calculation of some useful conduciveness figures for 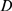 as per Equation (1). Using 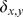 to denote Kronecker's delta function of the integers 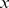 and 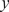, and 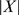 to denote the cardinality of set 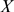, these statistics are:

- The distribution of full degrees in 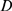, given by
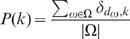
(4)for every possible full degree 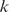.
- The distribution of depths in 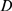, given by
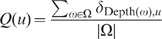
(5)for every possible depth 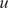.
- The distribution of widths in 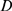, given by
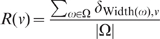
(6)for every possible width 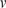.
- The joint distribution of full degrees and depths in 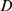, given by
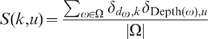
(7)for every possible full degree 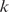 and depth 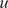.
- The joint distribution of full degrees and widths in 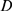, given by
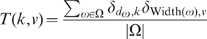
(8)for every possible full degree 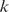 and width 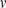.

Additional statistics are 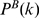, 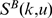, and 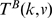, defined analogously to the above but for optimum-bound degrees (that is, substituting 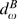 for 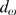 in the corresponding definitions). Note also that, whenever the 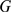 in question is one of the 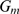 graphs of the sequence 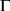 introduced previously, we alter the notation of these statistics by adopting the subscript 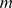 for them as well (consistently with the graph 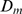 of node set 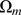 and edge set 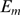, all introduced earlier but now with the specific meanings given in this section for 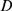, 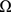, and 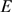, respectively).

## Results

**Figure 1:**
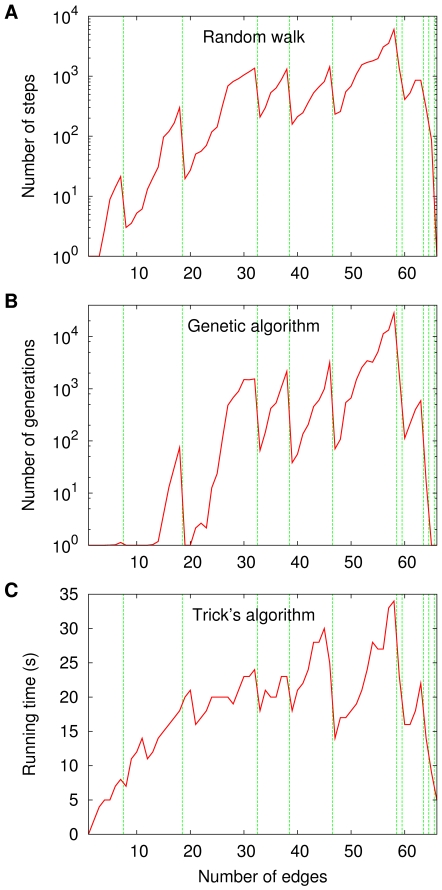
Performance of three algorithms to find the chromatic numbers of the graphs in 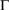. Data are given for a random walker (A), a genetic algorithm (B), and Trick's implementation [21] of the algorithm in [22] (C). The data shown for part A are averages over 10 000 independent runs. The genetic algorithm is the one described in the caption to Figure 15 in [7], parameter values included. It is based on viewing each acyclic orientation as an individual in a population and on letting selection favor those individuals that come closer to having optimal depth. It also relies on effecting acyclicity-preserving crossover and mutation operations. The data shown for part B are averages over 100 independent runs. Trick's algorithm assigns colors to nodes as a function of the colors their neighbors already have.

**Figure 2:**
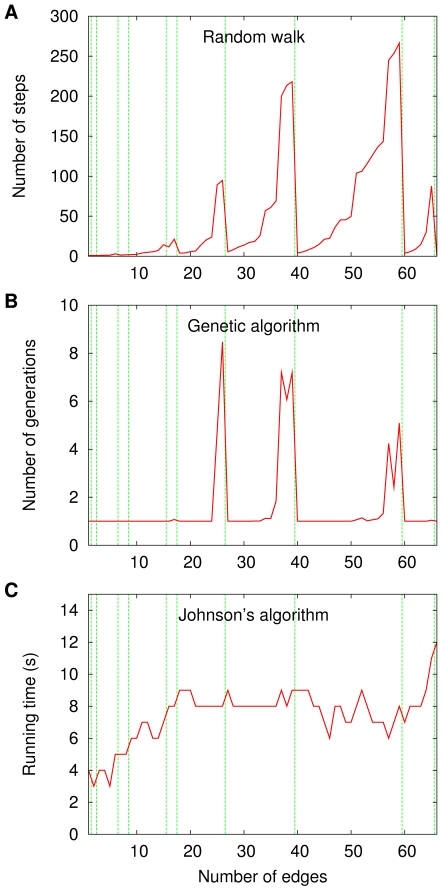
Performance of three algorithms to find the independence numbers of the graphs in 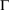. Data are given for a random walker (A), a genetic algorithm (B), and Johnson's implementation [23] of the algorithm in [24] (C). The data shown for part A are averages over 10 000 independent runs. The genetic algorithm is the one described in the caption to Figure 15 in [7], parameter values included. It is based on viewing each acyclic orientation as an individual in a population and on letting selection favor those individuals that come closer to having optimal width. It also relies on effecting acyclicity-preserving crossover and mutation operations. The data shown for part B are averages over 100 independent runs. Johnson's algorithm follows a simple branch-and-bound strategy.

Let us then look at one single sequence 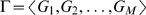 for 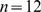 (whence 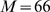) and observe how different algorithms to find 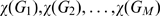 and 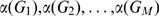 perform. Results on finding the chromatic numbers are given in Figure 1; those on finding the independence numbers are in Figure 2.

Figure 1 contains performance data on three algorithms. First is a simple random walker, which for each 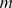 starts at a randomly chosen acyclic orientation in 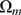 and then at each step traverses one of the edges that outgo from its current acyclic orientation in the set 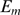, thus reaching another acyclic orientation. Performance data are then given for a genetic algorithm operating on 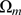, and then for a deterministic algorithm whose operation is not based on 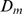 at all. The random walker and the genetic algorithm stop upon hitting the first acyclic orientation 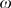 for which 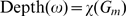, with the provision that 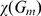 is known beforehand from running the third algorithm first. The data on all three algorithms are shown in the three parts of Figure 1 against a backdrop of vertical lines, each marking the critical number of edges right past which an increase in the chromatic number occurs: for 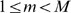, if 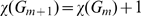, then a vertical line is drawn at the abscissa 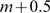. The chromatic numbers of the graphs in 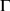 necessarily increase by 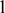 past each critical value, from 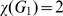 through 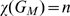, therefore there are 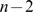 vertical lines all told.

A similar arrangement holds for Figure 2, whose setting differs from that of the previous one in that now both the random walker and the genetic algorithm stop upon finding 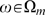 such that 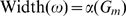, once again given that 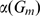 is known a priori from running the deterministic algorithm first. There is also an important difference regarding the changes in the graphs' independence numbers, which now necessarily decrease by 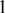 past each critical number of edges, from 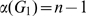 through 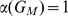, thus totaling 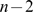 decreases as well. So, in Figure 2, the vertical lines marking the decreases are drawn at the abscissae 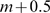 such that 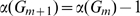.

**Figure 3:**
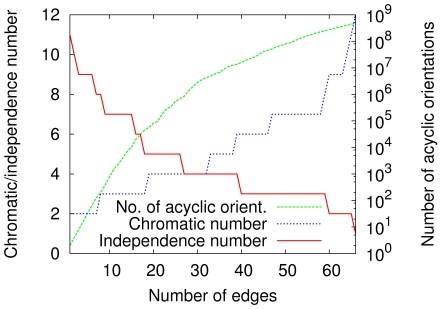
Chromatic- and independence-number variations at the transitions along 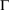. Chromatic-number increases and independence-number decreases occur by single units at the corresponding transitions, while the number of acyclic orientations grows steadily with the number of edges.

Given these critical numbers of edges, which depend on whether the graph-coloring or the independent-set problem is being addressed, we henceforth refer simply as a transition to the addition of one edge to the graph beyond some critical number. An explicit distinction between the two problems is made whenever needed for clarity. For the graph sequence 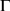, Figure 3 summarizes the stepwise growth of the chromatic number at the corresponding transitions, and similarly the diminution of the independence number. The figure also highlights the progress of the number of acyclic orientations as the number of edges increases. Clearly, the number of acyclic orientations grows rather rapidly and achieves the order of 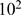 as early as the first transition related to the chromatic number.

Parts A and B of both Figures 1 and 2 refer to methods which work on the sets 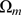 of acyclic orientations of the graphs 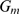, either making explicit use of the structure of each 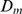 (which the random walker does) or allowing for longer jumps as the orientations undergo the crossover and mutation operations prescribed by the genetic algorithm. The data displayed in the corresponding four panels often have in common the property that the occurrence of a transition, say from 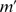 to 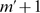 edges, causes the algorithm in question to perform significantly better on 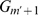 than on 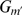, and then increasingly poorly through the following values of 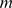 until the next transition, if any, is reached. This difference in performance is sometimes quite marked, involving improvements by at least one order of magnitude.

What is perhaps more curious is that the same behavior is also present in Figure 1C, which refers to a deterministic algorithm to find chromatic numbers that does not rely on the 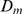 graphs (in fact, this algorithm's underlying strategy makes no reference at all to the acyclic orientations of the graph whose chromatic number it is seeking). Informally, then, this seems to indicate that the characteristic performance jumps at the transitions are inherent to the optimization problem itself (and only marginally, if at all, dependent upon how its feasible solutions are represented). It also seems to confer to the 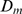 graphs some of the primitive representational character we sought in the beginning. However, Figure 2C, which also refers to a deterministic algorithm that does not operate on acyclic orientations, only now to find the graph's independence number, shows none of the effects on performance at the transitions that the random-walk and genetic-algorithm approaches exhibit. The reason for this is that, despite being just as nominally NP-hard as the problem of finding chromatic numbers, finding independence numbers is easier in practice than that other problem. What this means is that, in order for the transitions' effects on performance to show, substantially higher values of 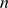 are needed (cf. Figure 9 in [7]).

We then proceed on the premise that the sequence of 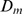 graphs for 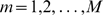 contains information enough to explain the performance jumps at the transitions, even though quantitatively we can only resort to what can be derived from each graph's nodes' depths, widths, and out-degrees. One initial indication that this makes sense comes from investigating the mutual information of pairs of random variables associated with each 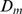. Given two discrete random variables and the joint distribution of their values, their mutual information is a measure of how much fixing the value of one of them reduces the uncertainty on the value of the other [20]. Just like Shannon's entropy, mutual information is expressed in (information-theoretic) bits.

In the context of finding 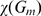, two discrete random variables of interest are those that give the out-degree and the depth of a randomly chosen node of 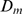. Their joint distribution is 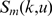 in the case of full degrees, 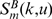 in the case of optimum-bound degrees, both introduced earlier. Their mutual information is, respectively for each case, given by
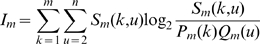
(9)and
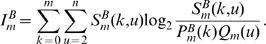
(10)If the problem is to find 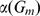, then the two random variables give the node's out-degree and its width. Their joint distributions are 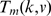 and 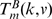, once again depending on whether full or optimum-bound degrees are referred to. The respective measures of mutual information are
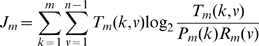
(11)and
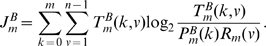
(12)


**Figure 4:**
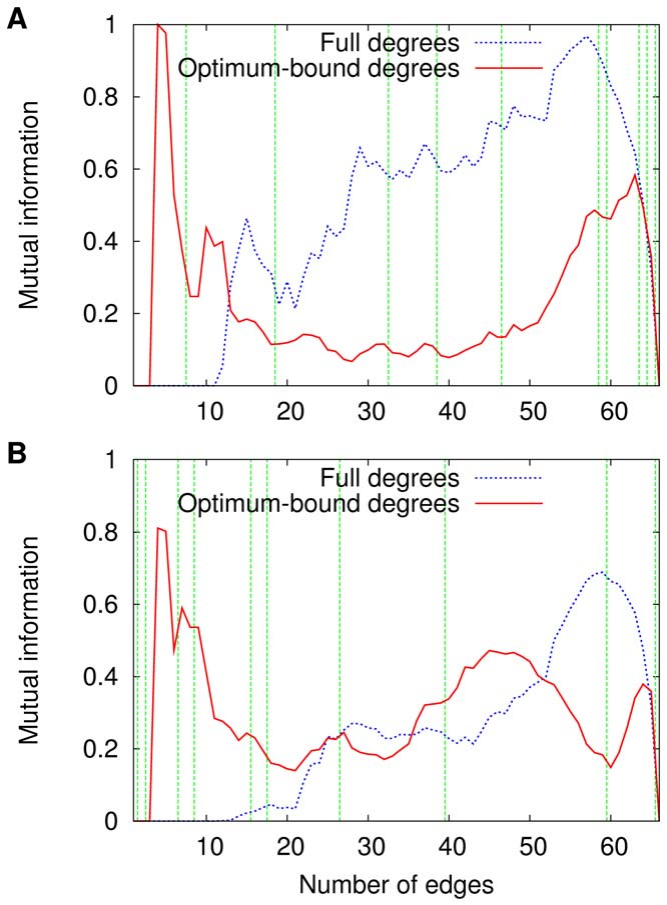
Evolution of the mutual information along 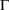. Data are given for 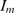 and 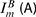 (A), which refer to coloring, respectively for full and optimum-bound degrees; and for 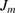 and 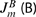 (B), which likewise refer to independent sets.

For the same sequence 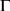 we have considered so far in this section, and following the same conventions as Figures 1 and 2 with regard to marking with vertical lines the values of 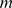 at which 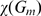 or 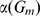 changes along the sequence, we show in Figure 4 the progress of these four mutual information functions, in part A for graph coloring, in part B for independent sets. Note, in all cases, that although consistently less than one bit, all four functions are nearly always positive, thus providing evidence that, in most 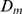 instances, out-degrees of either kind are independent from neither depths nor widths. However, the functions do not seem to behave consistently at the transitions and for this reason offer no direct explanation of what happens there.

It is important to recall that all of Figures 1, 2, 3, 4 refer to the one single sequence 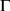. They have been offered as illustrations of what is typical, and averaging over multiple graph sequences, which requires that we address the fact that transitions may occur at different 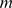 values in different sequences, might blur the reader's understanding of what the phenomenon is and how mutual information suggests that it has to do with the 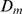 graphs. We now turn to the role played by graph conduciveness and, after one more single-sequence illustration, do some averaging as properly as possible.

Given a 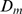 graph, let 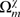 denote the subset of 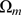 whose members are those of depth 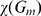. Analogously, let 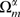 denote the subset of 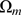 whose members are those of width 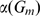. We study four kinds of conduciveness of 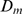, given as follows with reference to Equation (1); we use 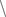
to denote set difference.
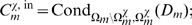
(13)

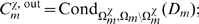
(14)

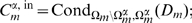
(15)

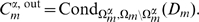
(16)Note that 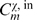 is the conduciveness of 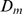 from all orientations that are non-optimal for coloring to those that are optimal, 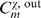 the conduciveness in the opposite direction. The situation with 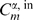 and 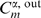 is totally analogous, now regarding optimality for independent sets. We refer to 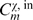 and 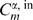 as being inbound, to 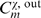 and 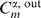 as being outbound. Note also that, by Equation (1), and given the antiparallel nature of the edge set of 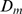, it holds that
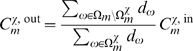
(17)and
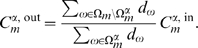
(18)These, however, imply no obvious relationship between the inbound conduciveness of 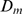 and its outbound conduciveness for any of the two problems.

**Figure 5:**
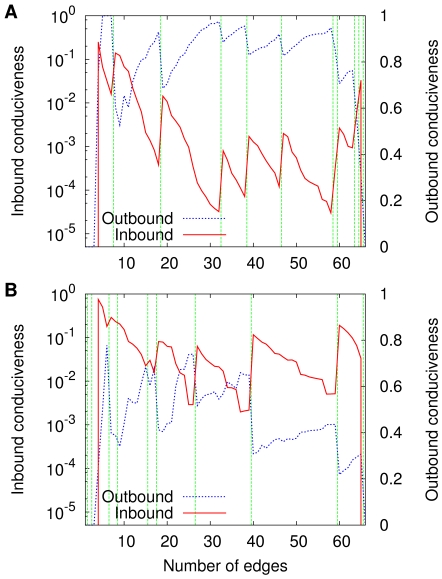
Evolution of network conduciveness along 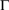. Data are given for 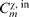 and 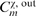 (A), which refer to coloring, respectively inbound and outbound; and for 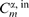 and 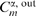 (B), which likewise refer to independent sets.

An illustration of the kind of relationship that does hold is given in Figure 5, which results from our last use of the same single sequence 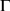 as heretofore. Strikingly, as the sequence unfolds with increasing 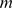 and the changes in 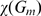 (part A of the figure) or 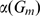 (part B of the figure) occur, the inbound conduciveness of 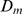 undergoes sudden jumps upwards precisely at the transitions while its outbound conduciveness undergoes downward jumps. The inbound-conduciveness jumps can be seen to encompass at least one order of magnitude in many cases. Between one transition and the next, the inbound conduciveness deteriorates progressively while the outbound conduciveness improves. This is then the key to interpreting the phenomena illustrated in Figures 1 and 2: at the transitions, 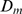 becomes markedly more conducive in the direction of the optimal orientations and less conducive in the opposite direction; right past a transition through right before the next one happens, 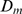 tends to become progressively less conducive in the direction of the optima, more conducive in the direction that leads away from them.

Next we study these conduciveness variations as averages over the graph sequences 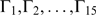, each comprising graphs on 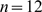 nodes and generated independently. As noted earlier, even though both the chromatic number and the independence number undergo 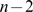 changes each at the transitions in each sequence, the 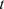
th transition, for some 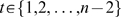, may happen at different values of 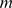 for the different sequences. Some alignment of the transitions is then needed for the averages of interest to be computed; we proceed as follows. If for a given sequence the 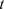
th transition related to the chromatic number occurs for 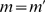, then we calculate the change ratios 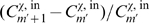 and 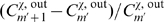. We do likewise for each transition related to the independence number. If two subsequent transitions related to the chromatic number occur at 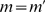 and 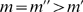, then we also calculate the change ratios 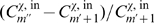 and 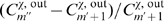, again proceeding likewise for the interval between every pair of subsequent transitions related to the independence number. The latter formulae can also be used to calculate change ratios for the interval that precedes the first transition (letting 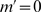 and 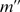 be the value of 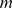 at which the first transition occurs) and the interval that succeeds the last transition (letting 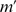 be value of 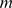 at which the last transition occurs and 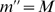). Once all change ratios have been calculated, they can be averaged over the 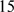 sequences for each transition (whichever the value of 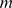 is at which it happens to occur in each sequence) and each interval.

**Figure 6:**
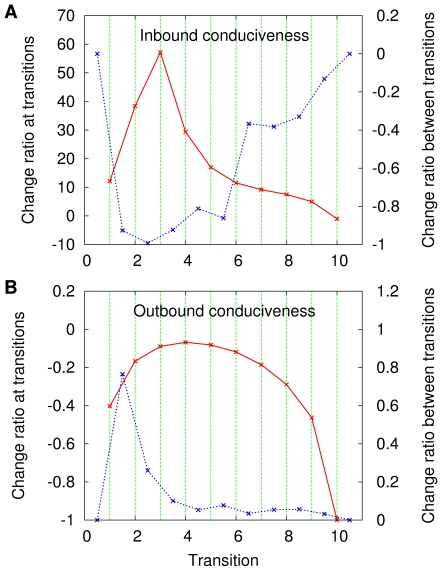
Conduciveness change ratios at the graph-coloring transitions and related intervals. Data are given as averages over the set of sequences 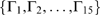 for both the inbound conduciveness (A) and the outbound conduciveness (B).

**Figure 7:**
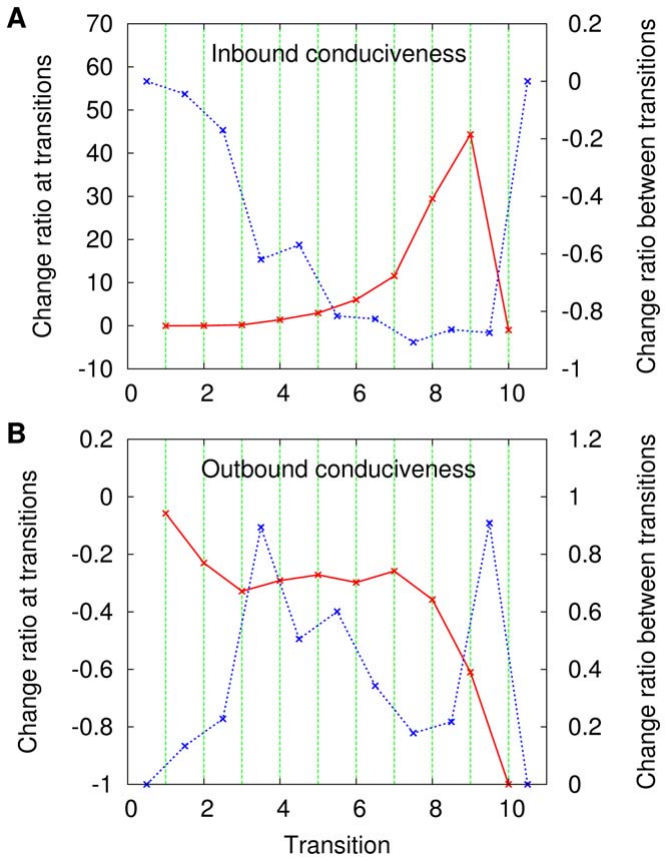
Conduciveness change ratios at the independent-set transitions and related intervals. Data are given as averages over the set of sequences 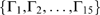 for both the inbound conduciveness (A) and the outbound conduciveness (B).

These average change ratios are given in Figures 6 and 7, respectively for graph coloring and independent sets. All data in these two figures are presented, as in most previous cases, against a backdrop of vertical lines. These, however, are now equally spaced and refer to the transition numbers, from 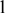 through 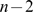, regardless of the 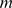 values at which the transitions themselves are observed in each particular sequence for each problem. Each panel in each figure contains two plots, one with points whose abscissae coincide with those of the vertical lines (this refers to change ratios at the transitions) and one with points whose abscissae stand either halfway between those of two consecutive vertical lines or to the left (right) of the leftmost (rightmmost) vertical line's abscissa [this refers to change ratios along the intervals between consecutive transitions or before (after) the first (last) transition].

Figures 6A and 7A, which refer to the progress of the inbound conduciveness as the transitions elapse, reveal that at most transitions the upward jumps represent significant fractions of the pre-transition conduciveness values, which often increase manyfold (by a factor of a few tens). On the other hand, the accumulated deterioration in conduciveness that is observed between transitions and in the outermost intervals is in most cases given by a fraction that varies widely depending on the transition, ranging practically from nearly no loss of the initial conduciveness inside the interval to nearly total loss.

Figures 6B and 7B, in turn, refer to how the outbound conduciveness values evolve along with the transitions and show that, right at the transitions, conduciveness is lost with respect to the pre-transition values by fractions that amount to losing from about 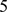
–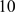
% of it (depending on the problem) to all of it. As we look at the accumulated improvement in conduciveness between transitions and in the outermost intervals, we see that a wide range of possibilities is again present, allowing at one extreme for practically no improvement and, at the other extreme, for an improvement by about 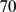
–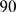
% of the initial conduciveness inside the interval (depending on the problem).

## Discussion

The notion of network conduciveness we have introduced is a simple degree-based indicator that can be interpreted as a probability with respect to a particular agent-related dynamics. We believe that, either as defined or as some variant thereof, it may find applications in network studies having to do with the dynamics of populations in networks. Our own application in this paper has been to the field of combinatorial optimization, and then the network in question is representative of the feasible solutions to a particular instance of an optimization problem and of how one may move from one solution to another through as simple a local transformation as possible. We tackled the NP-hard problems of finding an undirected graph's chromatic and independence numbers and demonstrated how network conduciveness, when applied to problem representations in the domain of the graph's acyclic orientations, is capable of helping explain the well-known performance jumps that occur along random sequences of graphs for both problems.

As it happens, though, the networks whose conduciveness we have considered grow very rapidly with the graph's numbers of nodes and edges, and become themselves very nearly intractable already for small instances of the problems we addressed. We were then limited in our computational experiments to using graphs on 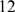 nodes exclusively and to averaging results on 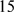 random sequences of graphs. For the sake of the record, with current technology all experiments required nearly two months on twenty processors. So, as much as we think that there is great potential usefulness to the notion of a network's conduciveness, further progress with the particular application we chose requires considerable further effort so that larger graphs and better statistical significance can be aimed at. On the other hand, we regard the first steps we have taken as very significant: to the best of our knowledge, no other study has addressed the intricacies of NP-hard optimization problems from the perspective of network theory applied to the structure that underlies the problems' sets of feasible solutions.
